# Supplementary material for: Experimentally Revealed Stochastic Preferences for Multicomponent Choice Options
Source: J Exp Psychol Anim Learn Cogn. 2020 Jul 27;46(4):367–84. doi: 10.1037/xan0000269 (PMC7547871; doi:10.1037/xan0000269)
Supplement: Supplementary file 1 [file XAN.docx]

**SUPPLEMENTARY MATERIAL**

**Supplementary Methods**

**Leave-one-out analysis of IPs.** We used a leave-one-out analysis to assess the meaningful representation of revealed preferences by the fitted ICs by testing the accuracy of the hyperbolic IC fit to the IPs. In this analysis, we removed one IP per IC (but not the initial Reference Bundle set at x = 0) and fitted an IC again with the same hyperbolic model as for the main IC fitting (see Methods, Eqs. 3, 3a). In total, for each original IC, we fitted 4 new ICs, each one leaving out a different IP. For each new IC, we assessed the deviation between the left-out IP and the refitted IC. We measured this deviation as difference of component B between the y-axis position of the original (but now left out) IP and the y-axis position of the refitted IC with the IP left out, at the same x-position (Figure S2B):

d = B_IP_ - B_refit_ Eq. S1

with d as difference (in ml; y-axis), B_IP_ as amount of component B of the left-out IP (ml), and B_refit_ as amount of component B on the refitted IC (ml). Thus, a difference of 0 ml suggested that removal of one IP did not affect the shape of the IC at all, thus indicating an excellent representation of revealed preferences by the fitted ICs, whereas any difference unequal to 0 ml quantified the degree of inaccuracy of this representation.

**Decoder analysis of preference levels.** To confirm the contribution of each IP to the two-dimensional representation of revealed preferences, we determined the accuracy (in percent correct) with which a randomly selected bundle, defined by the amounts of the two components A and B (in ml), could be assigned to its original revealed preference level as opposed to any one other level (binary distinction). By definition, each bundle that was psychophysically estimated to be as much revealed preferred as the Reference bundle constituted an IP; all bundles to which participants were choice indifferent against the same Reference Bundle constituted a series of IPs. In our experiment, three different Reference Bundles defined three preference levels (low, medium, high: component B: 2.0 ml, 5.0 ml or 8.0 ml, respectively; component A was always 0.0 ml; Figure S3A). The decoder used as inputs only bundles at the psychophysically estimated IPs (to which an IC was fitted using Eqs. 3, 3a), rather than bundles positioned on the fitted ICs.

Our main test employed a binary support vector machine (SVM) decoder separately on each individual participant. We used similar methods as previously described for predicting choice from neuronal activity (Tsutsui, Grabenhorst, Kobayashi, & Schultz, 2016). The SVM algorithm considered 5 IP bundles from each of 2 revealed preference levels (total of 10 IPs that had been assessed 12 times at each position in each participant) (Figure S3A). Each of the 2 preference levels was associated with a matrix of 2 columns (containing the x- and y-coordinates of bundle components A and B, respectively) and 5 rows (containing the 5 bundles). The 5 bundles were randomly selected (with replacement) from 60 bundles on each level (due to the random procedure with replacement, some bundles may have entered the algorithm multiple times, and not all five bundles may have been used for a given analysis). We left out 1 randomly selected bundle from the 10 bundles, trained the SVM algorithm with the remaining 9 bundles, and assessed whether the SVM decoder assigned the left-out bundle to its original revealed preference level or to another level. Thus we used 90% of the data for training the decoder and 10% for testing its classification performance. We repeated this procedure 10 times with the same selected 2 x 5 bundles but with a new randomly selected left-out bundle and calculated decoder accuracy as percent correct classification in these 10 trials. We repeated the random selection of the 2 x 5 bundles and the 10-trial accuracy assessment 150 times. For final decoding accuracy, we averaged the percentages from these 150 iterations (Table S3 left). We applied this procedure separately to all three possible combinations of two revealed preference levels (i. e. low and medium, medium and high, low and high). For assessing chance decoding, we shuffled the 2 x 5 matrix. Our earlier work has shown that increasing the number of analysis trials from 10 to 20 resulted in similar accuracy (Grabenhorst, Hernadi, & Schultz, 2016; Tsutsui, Grabenhorst, Kobayashi, & Schultz, 2016). The SVM was implemented with custom written software in Matlab R2015b (Mathworks) using the functions *svmtrain* and *svmclassify* with linear kernel (our previous work had shown that use of nonlinear kernels did not improve decoder performance; Tsutsui, Grabenhorst, Kobayashi, & Schultz, 2016).

We supplemented the SVM procedure with binary linear discriminant analysis (LDA) that provided visualization of the different levels of revealed preference (Figure S3). We used the same IPs and the same data matrices as for the SVM analysis (and the same IPs as used for the hyperbolic fitting of the three indifference curves, ICs). We obtained two variances; the discriminant 1 eigenvector captured the best separation between the three revealed preference levels as ‘between-level variance’ (colors in Figure S3); the discriminant 2 eigenvector captured the best within-level separation between five bundles on each of the three preference levels as ‘within-level variance’ (symbols in Figure S3). The results indicate visually the discrimination accuracy on the two axes of the two-dimensional plots. We also assessed the numeric accuracy of decoding as percent of correctly assigning a randomly selected bundle to its original revealed preference level. The decoder used the Matlab functions *fitcdiscr* and *predic* on z-normalised data from individual participants. For the LDA, our limited data required pooling from multiple participants. As revealed preferences are private and subjective, and therefore difficult to compare between individual participants, the LDA results should be considered as merely supportive and not as stand-alone data.

**Decoder analysis of BDM bids.** To assess the internal consistency of BDM bids, we used binary SVM analysis on bids from individual participants in analogy to SVM decoding of bundles according to preference levels. We tested the same IPs as used for hyperbolic IC fitting (Figure 4A). Each of the 2 preference levels was associated with a matrix of 1 column (containing the bids to each bundle) and 5 rows (containing the 5 bundles). The remainder of the bundle selection, repetition procedure and data shuffling was identical to that used for the SVM decoding for preference levels (see above). Thus, the SVM decoder for BDM bids assessed the accuracy with which the left-out bundle belonged to its original revealed preference level. We supplemented the SVM analysis of the BDM bids with analogous LDA for supportive visualization.

**Supplementary Results**

**Leave-one-out analysis of IPs.** We performed a leave-one-out analysis to assess the contribution of individual IPs to the ICs obtained from hyperbolic fits to the empirically estimated IPs in humans. We removed one IP at a time from the set of five IPs per IC (except the initial Reference Bundle at x = 0), and then refitted each IC with the remaining four IPs using the hyperbolic model, separately for each IC and each participant (total of 3 ICs x 24 participants = 72 ICs, with 4 IPs x 72 ICs = 288 IPs; see Supplementary Methods). We found consistency in the refitted ICs in four measures (Figure S2). First, none of the 72 refitted ICs overlapped with the refitted ICs at different levels in the same participant, thus demonstrating maintained IC separation despite one left-out IP. Second, none of the 72 refitted ICs overlapped with the 95% CIs of original ICs at different levels, confirming IC separation despite one left-out IP. Third, most refitted ICs (66 of 72 ICs, 92%) fell inside the 95% CIs of the original ICs, and the remainder curves (6 of 72 ICs, 8%) showed only some portions outside the 95% CIs of the original ICs, thus refuting possible overweighted influence of individual IPs on ICs. Fourth, numeric comparisons showed only insignificant deviations between refitted ICs and the IPs that had been left out when refitting the curves (vertical distance of 0.05 ± 0.13 ml in all 24 participants; mean ± standard error of the mean, SEM; *N* = 336; *P =* 0.98 against normal distribution; t-test), confirming absence of overweighted IP influence on ICs. These four results suggest that the hyperbolically fitted ICs captured the IPs consistently and provided valid representations of the revealed preferences.

**Decoder analysis of preference levels.** We used a single-dimensional linear support vector machine (SVM) decoder as different statistical procedure to confirm the contribution of each IP to the two-dimensional representation of revealed preferences by ICs. In each participant, we set a given test bundle to one of the psychophysically determined IPs (Figure S3A) and assessed the accuracy with which the decoder assigned that bundle to its original preference level (each preference level was defined by a series of empirically estimated IPs but was not a fitted IC). SVM decoding accuracy ranged largely from 70% to 100% (*P* = 2.055 x 10^-101^), although a few lower values were observed (Table S3 left); shuffled data failed to discriminate between preference levels (accuracies of 44.7% - 54.6%; Table S4 left).

We supplemented the SVM analysis by visualization of decoding using two-dimensional linear discriminant analysis (LDA). The considerable amount of data necessary for reasonable LDA required us to pool data from several participants, which violates a basic tenet of economic theory that prohibits pooling of subjective preferences across individual participants. To somewhat contain expected inaccuracies, we normalised IPs across participants (z-score normalization for reward B along the y-axis; reward A had been experimentally set to identical values on the x-axis) and restricted the analysis to specific subsets of participants. The LDA confirmed the SVM results in all participant subsets (Figure S3); the first linear discriminant assigned bundles to the three revealed preference levels, as shown by spatial separation of the three colored groups, with a numeric accuracy of 80-100% (*P* = 1.148 x 10^-97^). By contrast, the second discriminant failed to accurately assign bundles to different positions on same preference levels, as shown by the mix of the five shapes representing bundle position. These characteristics were seen in six participants whose fitted ICs showed the highest similarity in convexity (Figure S3B, C), in six participants with linear ICs (Figure S3D, E) and, for comparison, in all 24 participants (Figure S3F, G) (for distinction of participants based on IC curvature, see two highest bars in Figure 2F). Thus, the two-dimensional LDA decoding followed the fundamentals of ICs: preference for bundles on higher ICs but indifference along ICs.

Taken together, the two decoders confirmed three distinguishable levels of IPs, and LDA in addition confirmed indifference between IPs on same levels. As these IPs constituted the basis for hyperbolic fitting of the three ICs, the decoder results validated also the fitting procedure and confirmed the representation of revealed preferences by the empirically estimated ICs.

**Decoder analysis of BDM bids.** We used decoders to test the distinction of bundle position between but not along preference levels. Using BDM bids, a binary SVM decoder showed good accuracy of assigning a test bundle to its original preference level in individual participants (mostly 50-70%; *P* = 3.789 x 10^-9^; Table S3 right) but not with shuffled data (45.8% - 54.7%; Table S4 right. We used an LDA to decode several levels together and found good visual assignment of the test bundles to the three revealed preference levels in our combined population of 24 participants (first discriminant; numeric accuracy of 88-100%; *P* = 9.46 x 10^-12^; Figure S4; three colored symbol groups) but not to different positions on same preference levels (second discriminant; numeric accuracy of 43-51%; *P* = 0.1433; mix of shapes) (note the reservations above when combining data from multiple participants). Thus, the two decoders showed together that the participants' BDM bids distinguished bundles well between preference levels but not on the same preference level, thus confirming BDM validation of the two-dimensional revealed preference scheme of the ICs.

*Table S1A.* Test amounts (ml) of milkshake component A for stepwise psychophysical assessment of choice indifference points (IP): lowest indifference curve (IC1).

| Participant | Step 1 | Step 2 | Step 3 | Step 4 | Step 5 |
| --- | --- | --- | --- | --- | --- |
| 1 | 0.000 | 0.053 | 0.155 | 0.395 | 0.870 |
| 2 | 0.000 | 0.147 | 0.320 | 0.526 | 0.769 |
| 3 | 0.000 | 0.149 | 0.312 | 0.492 | 0.690 |
| 4 | 0.000 | 0.184 | 0.442 | 0.796 | 1.250 |
| 5 | 0.000 | 0.194 | 0.392 | 0.594 | 0.800 |
| 6 | 0.000 | 0.070 | 0.236 | 0.685 | 1.333 |
| 7 | 0.000 | 0.070 | 0.228 | 0.634 | 1.250 |
| 8 | 0.000 | 0.161 | 0.371 | 0.647 | 1.000 |
| 9 | 0.000 | 0.113 | 0.283 | 0.548 | 0.939 |
| 10 | 0.000 | 0.067 | 0.186 | 0.428 | 0.870 |
| 11 | 0.000 | 0.033 | 0.104 | 0.334 | 0.870 |
| 12 | 0.000 | 0.009 | 0.027 | 0.436 | 1.176 |
| 13 | 0.000 | 0.058 | 0.171 | 0.444 | 0.952 |
| 14 | 0.000 | 0.157 | 0.358 | 0.619 | 0.952 |
| 15 | 0.000 | 0.116 | 0.265 | 0.460 | 0.714 |
| 16 | 0.000 | 0.046 | 0.130 | 0.315 | 0.714 |
| 17 | 0.000 | 0.194 | 0.392 | 0.594 | 0.800 |
| 18 | 0.000 | 0.069 | 0.175 | 0.355 | 0.667 |
| 19 | 0.000 | 0.053 | 0.155 | 0.395 | 0.870 |
| 20 | 0.000 | 0.216 | 0.490 | 0.833 | 1.250 |
| 21 | 0.000 | 0.089 | 0.216 | 0.405 | 0.690 |
| 22 | 0.000 | 0.143 | 0.304 | 0.485 | 0.690 |
| 23 | 0.000 | 0.089 | 0.216 | 0.405 | 0.690 |
| 24 | 0.000 | 0.044 | 0.122 | 0.285 | 0.645 |
| MEAN | 0.000 | 0.105 | 0.252 | 0.504 | 0.894 |
| SEM | 0.000 | 0.012 | 0.024 | 0.030 | 0.044 |

Step 1: Reference Bundle set to (x = 0.0 ml; y = 2.0 ml). Steps 1- 5: test amounts of component A (x-axis) of the Variable Bundle for psychophysical variation of component B (y-axis).

*Table S1B.* Test amounts (ml) of milkshake component A for stepwise psychophysical assessment of choice indifference points (IP): intermediate indifference curve (IC2).

| Participant | Step 1 | Step 2 | Step 3 | Step 4 | Step 5 |
| --- | --- | --- | --- | --- | --- |
| 1 | 0.000 | 0.135 | 0.391 | 0.990 | 2.174 |
| 2 | 0.000 | 0.359 | 0.788 | 1.303 | 1.923 |
| 3 | 0.000 | 0.268 | 0.618 | 1.088 | 1.724 |
| 4 | 0.000 | 0.176 | 0.569 | 1.585 | 3.125 |
| 5 | 0.000 | 0.426 | 0.897 | 1.420 | 2.000 |
| 6 | 0.000 | 0.132 | 0.482 | 1.646 | 3.333 |
| 7 | 0.000 | 0.165 | 0.544 | 1.567 | 3.125 |
| 8 | 0.000 | 0.446 | 0.996 | 1.674 | 2.500 |
| 9 | 0.000 | 0.231 | 0.610 | 1.278 | 2.347 |
| 10 | 0.000 | 0.161 | 0.450 | 1.051 | 2.174 |
| 11 | 0.000 | 0.025 | 0.094 | 0.605 | 2.174 |
| 12 | 0.000 | 0.070 | 0.270 | 1.260 | 2.941 |
| 13 | 0.000 | 0.139 | 0.415 | 1.094 | 2.381 |
| 14 | 0.000 | 0.269 | 0.686 | 1.362 | 2.381 |
| 15 | 0.000 | 0.136 | 0.370 | 0.842 | 1.786 |
| 16 | 0.000 | 0.179 | 0.460 | 0.944 | 1.786 |
| 17 | 0.000 | 0.426 | 0.897 | 1.420 | 2.000 |
| 18 | 0.000 | 0.143 | 0.378 | 0.819 | 1.667 |
| 19 | 0.000 | 0.135 | 0.391 | 0.990 | 2.174 |
| 20 | 0.000 | 0.534 | 1.216 | 2.077 | 3.125 |
| 21 | 0.000 | 0.185 | 0.467 | 0.935 | 1.724 |
| 22 | 0.000 | 0.318 | 0.699 | 1.161 | 1.724 |
| 23 | 0.000 | 0.185 | 0.467 | 0.935 | 1.724 |
| 24 | 0.000 | 0.134 | 0.357 | 0.779 | 1.613 |
| MEAN | 0.000 | 0.224 | 0.563 | 1.201 | 2.234 |
| SEM | 0.000 | 0.027 | 0.052 | 0.071 | 0.109 |

Conventions as for Table S1A, but Reference Bundle (Step 1) set to (x = 0.0 ml; y = 5.0 ml).

*Table S1C.* Test amounts (ml) of milkshake component A for stepwise psychophysical assessment of choice indifference points (IP): highest indifference curve (IC3).

| Participant | Step 1 | Step 2 | Step 3 | Step 4 | Step 5 |
| --- | --- | --- | --- | --- | --- |
| 1 | 0.000 | 0.299 | 0.809 | 1.781 | 3.478 |
| 2 | 0.000 | 0.535 | 1.199 | 2.031 | 3.077 |
| 3 | 0.000 | 0.552 | 1.185 | 1.915 | 2.759 |
| 4 | 0.000 | 0.402 | 1.174 | 2.732 | 5.000 |
| 5 | 0.000 | 0.630 | 1.361 | 2.210 | 3.200 |
| 6 | 0.000 | 0.281 | 0.948 | 2.745 | 5.333 |
| 7 | 0.000 | 0.394 | 1.155 | 2.714 | 5.000 |
| 8 | 0.000 | 0.637 | 1.473 | 2.577 | 4.000 |
| 9 | 0.000 | 0.492 | 1.203 | 2.256 | 3.756 |
| 10 | 0.000 | 0.244 | 0.689 | 1.649 | 3.478 |
| 11 | 0.000 | 0.035 | 0.129 | 0.933 | 3.478 |
| 12 | 0.000 | 0.096 | 0.380 | 1.976 | 4.706 |
| 13 | 0.000 | 0.323 | 0.886 | 1.974 | 3.810 |
| 14 | 0.000 | 0.397 | 1.035 | 2.121 | 3.810 |
| 15 | 0.000 | 0.212 | 0.581 | 1.334 | 2.857 |
| 16 | 0.000 | 0.292 | 0.747 | 1.522 | 2.857 |
| 17 | 0.000 | 0.630 | 1.361 | 2.210 | 3.200 |
| 18 | 0.000 | 0.134 | 0.391 | 1.029 | 2.667 |
| 19 | 0.000 | 0.299 | 0.809 | 1.781 | 3.478 |
| 20 | 0.000 | 0.844 | 1.929 | 3.311 | 5.000 |
| 21 | 0.000 | 0.353 | 0.856 | 1.610 | 2.759 |
| 22 | 0.000 | 0.445 | 1.016 | 1.766 | 2.759 |
| 23 | 0.000 | 0.353 | 0.856 | 1.610 | 2.759 |
| 24 | 0.000 | 0.189 | 0.514 | 1.175 | 2.581 |
| MEAN | 0.000 | 0.378 | 0.945 | 1.957 | 3.575 |
| SEM | 0.000 | 0.039 | 0.081 | 0.119 | 0.175 |

Conventions as for Table S1A, but Reference Bundle (Step 1) set to (x = 0.0 ml; y = 8.0 ml).

*Table S2.* Amounts of milkshake component B (ml; y-axis) at the 7 test points for psychophysical estimation of indifference points for the lowest, intermediate and highest indifference curves (IC1 - IC3).

| Test point | 1 | 2 | 3 | 4 | 5 | 6 | 7 |
| --- | --- | --- | --- | --- | --- | --- | --- |
| IC1 | 0.000 | 0.333 | 0.667 | 1.000 | 1.333 | 1.667 | 2.000 |
| IC2 | 0.000 | 0.833 | 1.667 | 2.500 | 3.333 | 4.167 | 5.000 |
| IC3 | 0.000 | 1.333 | 2.667 | 4.000 | 5.333 | 6.667 | 8.000 |

*Table S3.* Accuracy (in %) of assigning bundles and BDM bids to original preference levels, using support vector machine decoder.

|  | Bundles | | | BDM bids | | |
| --- | --- | --- | --- | --- | --- | --- |
| Participant  C – convex IC  L – linear IC | Lev1  vs.  Lev2 | Lev1  vs.  Lev3 | Lev2  vs.  Lev3 | Lev1  vs.  Lev2 | Lev1  vs.  Lev3 | Lev2  vs.  Lev3 |
| C1 | 83.9 | 100.0 | 68.8 | 70.6 | 81.0 | 54.4 |
| C2 | 86.8 | 99.8 | 68.7 | 44.0 | 51.0 | 47.4 |
| C3 | 72.7 | 96.0 | 58.7 | 56.6 | 71.3 | 59.3 |
| C4 | 88.0 | 99.5 | 67.6 | 67.7 | 76.3 | 62.5 |
| C5 | 99.1 | 100.0 | 81.4 | 64.8 | 69.9 | 49.8 |
| C6 | 93.7 | 99.5 | 61.9 | 53.5 | 65.9 | 54.0 |
| C7 | 66.2 | 75.5 | 49.5 | 70.0 | 72.2 | 43.6 |
| C8 | 72.9 | 81.8 | 52.6 | 49.8 | 67.2 | 62.6 |
| C9 | 80.0 | 100.0 | 80.0 | 68.4 | 83.8 | 62.1 |
| C10 | 99.6 | 100.0 | 69.8 | 47.0 | 46.6 | 43.7 |
| C11 | 90.2 | 99.5 | 55.4 | 68.9 | 75.7 | 53.1 |
| C12 | 96.8 | 100.0 | 71.2 | 47.3 | 57.9 | 47.1 |
| C12 | 86.1 | 87.7 | 56.7 | 58.7 | 60.9 | 48.2 |
| C14 | 80.0 | 100.0 | 80.0 | 63.2 | 74.3 | 56.8 |
| C15 | 99.8 | 100.0 | 85.5 | 67.8 | 78.6 | 54.8 |
| C16 | 100.0 | 100.0 | 90.0 | 57.8 | 62.5 | 50.4 |
| C17 | 100.0 | 100.0 | 100.0 | 44.8 | 70.9 | 68.4 |
| C18 | 99.0 | 99.9 | 55.3 | 68.1 | 75.3 | 51.7 |
| L19 | 100.0 | 100.0 | 88.6 | 43.5 | 65.8 | 59.6 |
| L20 | 100.0 | 100.0 | 95.9 | 69.2 | 84.9 | 70.1 |
| L21 | 100.0 | 100.0 | 100.0 | 62.2 | 66.0 | 44.4 |
| L22 | 100.0 | 100.0 | 83.6 | 54.8 | 52.5 | 45.9 |
| L23 | 100.0 | 100.0 | 100.0 | 63.2 | 58.4 | 47.8 |
| L24 | 100.0 | 100.0 | 80.0 | 61.4 | 68.5 | 57.7 |

Accuracy is shown in % of numbers of bundles and BDM bids correctly assigned to one of the two tested revealed preference levels (averages from 150 iterations of classification of 10 pseudorandomly selected bundles / BDM bids from two tested revealed preference levels). Bundles were tested at psychophysically assessed choice indifference points (IP) on one of three revealed preference levels (corresponding to the three indifference curves, IC, fitted to the IPs). The tested bundles had been pseudorandomly selected from 5 unique estimated IPs on each of two revealed preference levels under study. BDM: Becker-DeGroot-Marschak auction-like bidding mechanism. Lev1, Lev2, Lev3: revealed preference levels, numbered according to distance from origin. The 24 participants were labelled as C or L according to their IPs being fitted best to convex or linear ICs.

*Table S4.* Chance decoding accuracy of shuffled data by support vector machine.

|  | Bundles | | | BDM bids | | |
| --- | --- | --- | --- | --- | --- | --- |
| Participant  C – convex IC  L – linear IC | Lev1  vs.  Lev2 | Lev1  vs.  Lev3 | Lev2  vs.  Lev3 | Lev1  vs.  Lev2 | Lev1  vs.  Lev3 | Lev2  vs.  Lev3 |
| C1 | 53.5 | 50.9 | 51.3 | 45.8 | 49.1 | 52.0 |
| C2 | 49.6 | 48.9 | 50.0 | 50.1 | 51.7 | 53.2 |
| C3 | 50.2 | 52.5 | 51.0 | 50.3 | 50.2 | 50.8 |
| C4 | 48.6 | 48.2 | 52.0 | 53.1 | 50.2 | 50.1 |
| C5 | 48.8 | 51.8 | 51.2 | 51.6 | 50.7 | 50.0 |
| C6 | 46.8 | 50.3 | 53.2 | 48.4 | 49.2 | 50.2 |
| C7 | 48.3 | 51.3 | 49.2 | 49.7 | 50.2 | 49.7 |
| C8 | 48.9 | 48.9 | 49.2 | 50.2 | 51.6 | 50.1 |
| C9 | 50.3 | 52.0 | 46.9 | 52.0 | 50.0 | 49.9 |
| C10 | 49.7 | 52.4 | 49.4 | 47.2 | 53.0 | 51.4 |
| C11 | 48.6 | 52.2 | 44.7 | 49.2 | 50.4 | 49.6 |
| C12 | 49.5 | 53.1 | 46.9 | 52.3 | 48.7 | 50.5 |
| C12 | 49.6 | 48.9 | 49.5 | 50.3 | 53.1 | 50.3 |
| C14 | 52.1 | 51.7 | 51.3 | 52.3 | 52.0 | 50.4 |
| C15 | 49.2 | 53.0 | 47.2 | 52.4 | 49.5 | 47.7 |
| C16 | 53.4 | 52.5 | 47.2 | 46.5 | 51.1 | 47.1 |
| C17 | 49.8 | 53.0 | 50.7 | 52.9 | 52.0 | 51.0 |
| C18 | 49.8 | 48.2 | 52.0 | 51.4 | 48.4 | 49.9 |
| L19 | 48.4 | 53.7 | 52.6 | 50.0 | 50.7 | 47.0 |
| L20 | 50.0 | 53.4 | 49.8 | 49.7 | 54.1 | 52.2 |
| L21 | 54.6 | 53.4 | 48.5 | 54.2 | 52.4 | 54.7 |
| L22 | 47.0 | 52.7 | 51.1 | 51.9 | 48.9 | 47.7 |
| L23 | 51.4 | 53.1 | 47.2 | 50.4 | 51.2 | 50.1 |
| L24 | 49.3 | 50.3 | 48.6 | 49.7 | 49.9 | 49.5 |

The support vector machine analysis used the shuffled matrix of 2 columns (containing the x- and y-coordinates of bundle components A and B, respectively) and 5 rows (containing the 5 bundles). Conventions as for Table S4.

*Table S5.* Two-way Analysis of Variance (ANOVA) of BDM bids.

|  | 1st factor F(2,179) | | | 2nd factor F(4,179) | | | Interaction F(8,179) | | |
| --- | --- | --- | --- | --- | --- | --- | --- | --- | --- |
| Participant | P | F | MSE | P | F | MSE | P | F | MSE |
| 1* | 9.98 x 10^-46^ | 128.68 | 1656.30 | 0.01* | 2.07 | 26.70 | 0.99 | 0.45 | 5.78 |
| 2 | 5.37 x 10^-40^ | 109.15 | 1829.07 | 0.53 | 0.93 | 15.51 | 1.00 | 0.34 | 5.74 |
| 3 | 5.63 x 10^-63^ | 194.16 | 2148.07 | 0.61 | 0.86 | 9.47 | 0.70 | 0.84 | 9.32 |
| 4* | 5.68 x 10^-79^ | 265.03 | 2585.34 | 0.02* | 1.99 | 19.37 | 0.90 | 0.67 | 6.50 |
| 5* | 3.53 x 10^-56^ | 167.10 | 2300.07 | 0.03* | 1.84 | 25.28 | 0.71 | 0.84 | 11.51 |
| 6 | 2.13 x 10^-49^ | 141.75 | 1773.60 | 0.64 | 0.83 | 10.39 | 0.96 | 0.58 | 7.30 |
| 7 | 8.67 x 10^-41^ | 111.79 | 1620.12 | 0.83 | 0.64 | 9.28 | 1.00 | 0.20 | 2.85 |
| 8 | 4.25 x 10^-58^ | 174.57 | 1793.40 | 0.97 | 0.41 | 4.24 | 1.00 | 0.21 | 2.18 |
| 9 | 8.04 x 10^-60^ | 181.39 | 1716.27 | 0.98 | 0.39 | 3.65 | 1.00 | 0.14 | 1.30 |
| 10 | 9.28 x 10^-34^ | 89.04 | 1656.30 | 0.80 | 0.68 | 12.63 | 1.00 | 0.27 | 5.10 |
| 11 | 9.81 x 10^-53^ | 154.02 | 1681.34 | 0.88 | 0.58 | 6.31 | 1.00 | 0.26 | 2.79 |
| 12 | 5.38 x 10^-116^ | 475.79 | 4001.40 | 0.98 | 0.38 | 3.22 | 1.00 | 0.21 | 1.78 |
| 13 | 6.88 x 10^-49^ | 139.91 | 1956.07 | 0.38 | 1.08 | 15.07 | 0.99 | 0.46 | 6.37 |
| 14 | 1.27 x 10^-65^ | 205.17 | 2160.00 | 0.93 | 0.51 | 5.39 | 1.00 | 0.19 | 2.04 |
| 15 | 5.86 x 10^-63^ | 194.09 | 2527.41 | 0.79 | 0.69 | 8.97 | 1.00 | 0.34 | 4.37 |
| 16 | 7.10 x 10^-52^ | 150.82 | 2383.27 | 0.77 | 0.70 | 11.09 | 0.99 | 0.49 | 7.68 |
| 17 | 1.82 x 10^-74^ | 243.99 | 2574.67 | 0.69 | 0.78 | 8.20 | 1.00 | 0.19 | 1.99 |
| 18 | 2.81 x 10^-70^ | 225.21 | 2667.23 | 0.68 | 0.79 | 9.40 | 0.99 | 0.48 | 5.72 |
| 19 | 2.13 x 10^-48^ | 138.15 | 2005.07 | 0.83 | 0.64 | 9.27 | 0.99 | 0.45 | 6.52 |
| 20 | 4.82 x 10^-58^ | 174.35 | 1865.36 | 0.87 | 0.59 | 6.32 | 1.00 | 0.25 | 2.69 |
| 21 | 1.61 x 10^-72^ | 235.16 | 2486.40 | 0.43 | 1.02 | 10.82 | 1.00 | 0.28 | 2.99 |
| 22 | 2.67 x 10^-56^ | 167.56 | 1658.40 | 0.07 | 1.63 | 16.15 | 0.60 | 0.91 | 8.98 |
| 23 | 4.61 x 10^-108^ | 424.33 | 4768.92 | 0.05 | 1.70 | 19.15 | 0.37 | 1.07 | 12.07 |
| 24 | 1.49 x 10^-73^ | 239.83 | 2029.07 | 0.39 | 1.06 | 9.00 | 0.40 | 1.05 | 8.89 |

F(df1, df2): 1st factor: df1 = 2 (n - 1, n = 3 preference levels), df2 = 179 (n - 1, n = 5 IPs x 3 levels x 12 trial repetitions = 180); 2nd factor: df1 = 4 (n - 1, n = 5 IPs per preference level), df2 = 179; Interaction: df1 = 8 ((n - 1 ) x (m - 1), n = 3 preference levels, m = 5 IPs per preference level), df2 = 179. df: degree of freedom; IP: bundle at indifference point; MSE: mean square error; * Exceptional 3 participants with significantly different BDM bids in 2nd factor (*P* < 0.05).

*Figure S1.* Empirical indifference curves (IC) from all 24 participants. Note the fanning out of the confidence intervals towards the bottom right in each graph (thin lines), which likely reflects the progression of choice testing: the Reference Bundle was kept constant at the y-axis intercept (x = 0), whereas testing with the Variable Bundle progressed from top left to bottom right. Same conventions as for Figure 2.

*Figure S2.* Leave-one-out validation of estimated indifference curves (ICs).

(A) Graphic assessment with an example participant: hyperbolically refitted ICs with one left-out indifference point (IP) (solid lines), plotted together with 95% confidence intervals of the original hyperbolically fitted ICs (dotted lines). The refitting resulted in 4 new ICs (partly overlapping) at each of three levels. None of the refitted IPs fell outside the original confidence intervals.

(B) Scheme of numeric assessment: distance in ml (in ml on y-axis, red) between the left-out IP on the original IC (heavy black dot on black curve) and the refitted IC (green).

(C) Histogram of distance between refitted ICs and left-out IPs across all subjects. Skewness was -0.12, suggesting rather symmetric distribution around the mean (modeled in red).

*Figure S3.* Visualization of bundle decoding using Linear Discriminant Analysis (LDA).

(A) Schematics of bundle decoding at psychophysically estimated points of equal revealed preference (indifference points, IPs, plotted along the dotted lines). Following the notions of revealed preference, LDA should show accurate decoding between the three preference levels (green, blue, red) but not along each level.

(B) LDA bundle distinction between three levels of revealed preference (81-100% binary numeric decoding accuracy between two levels; first discriminant) but not along same preference levels (second discriminant) in N = 80 bundles from six participants (6P) with similar convex ICs (same convention applies to all panels). Bundles on the three preference levels are colored blue, green and red according to distance from origin, red being highest. The five bundles on each preference level are marked from top left to bottom right with ‘o’, ‘*’, ‘+’, ‘x’ and ‘□’ symbols. Due to the arbitrariness of the scale, numbers are not indicated.

(C) As B but for partly physically non-dominating bundles (one lower component in preferred bundle than in alternative bundle (97-100% accuracy; first discriminant).

(D) As B but for six participants with linear ICs (90-100% accuracy).

(E) As D but for partly physically non-dominating bundles (96-100% accuracy).

(F) As B but for all 24 participants (18 with convex IC, six with linear ICs) (83-100% accuracy).

(G) As F but for partly physically non-dominating bundles (97-100% accuracy).

*Figure 4.* Visual decoding by Linear Discriminant Analysis (LDA) of BDM bids for bundles on different revealed preference levels (first discriminant; 88-100% numeric decoding accuracy; *P* = 9.46 x 10^-12^) and along same preference levels (second discriminant; 43-51% accuracy) (N = 243 bundles; all 24 participants). The LDA used scalar BDM bids for bundles positioned at IPs shown in Figure 4A.
